# Exploring lived experiences in home-based psychiatric care: a qualitative study of service users, families, and professionals in Spain

**DOI:** 10.3389/fpsyt.2025.1670470

**Published:** 2025-09-23

**Authors:** Ana María Besoaín-Cornejo, Montserrat Gil-Girbau, Mariam Alouali-Moussakhkhar, Luisa Baladón Higueras, Josefina Sáez, Maria Rubio-Valera

**Affiliations:** 1 Health Technology Assessment in Primary Care and Mental Health (PRISMA) Research Group, Teaching, Research & Innovation Unit, Institut de Recerca Sant Joan de Déu, Esplugues de Llobregat, Spain; 2 Network for Research on Chronicity, Primary Care and Health Promotion (RICAPPS), Instituto de Salud Carlos III, Madrid, Spain; 3 Facultat de Farmàcia i Ciències de l’Alimentació, Universitat de Barcelona (UB), Barcelona, Spain; 4 Parc Sanitari Sant Joan de Déu, Sant Boi de Llobregat, Spain; 5 Patient Research Partner, Sant Pere de Ribes, Barcelona, Spain; 6 Center for Biomedical Research Network in Epidemiology and Public Health (CIBERESP), Instituto de Salud Carlos III, Madrid, Spain; 7 Medicine and Life Sciences, Universitat Pompeu Fabra, Barcelona, Spain

**Keywords:** crisis management, health services research, mental health, patient-centered care, qualitative research

## Abstract

**Introduction:**

Psychiatric home care provides a wide range of multidisciplinary, user-centered, high-intensity psychiatric interventions to manage mental health crises. Previous studies have found high satisfaction with care, but only assessed user and nurse perspectives. This study aimed to understand the experience of a psychiatric home hospitalization program in Spain from the perspective of all involved (users, families and healthcare professionals).

**Materials and methods:**

A qualitative study with a phenomenological approach was conducted to assess “Crisis Resolution and Home Treatment” (CRHT), a psychiatric home care program. Semi-structured individual and group interviews were held between 2021 and 2022. The final sample size was determined by saturation and data were analyzed thematically. Analyses were performed by a multidisciplinary team and externally reviewed by a mental health user and an experienced CRHT manager.

**Results:**

Four main themes summarizing CRHT experiences emerged: (1) Organizing and operating CRHT programs; (2) Receiving care at home; (3) Caregiver and family involvement and (4) Consequences of the home-based care model. CRHT allows individualized and contextualized treatment. Users and families valued home care and felt safe, although unprepared for the post-discharge situation, especially as care intensity decreases. While home care strengthens family bonds, some caregivers may need additional support to manage a crisis. Along with personalized care, CRHT allows for the development of a comprehensive lifelong treatment plan, although the care burden (for oneself and others) must be considered.

**Conclusion:**

CRHT was rated positively as a flexible intervention, facilitating person-centered care and strengthening trust between users, their families, and the CRHT team. It offers personalized treatment and connects individuals to further support, enabling better treatment experiences and strengthening family relationships.

## 1 Introduction

Psychiatric home-based hospitalization or psychiatric home care typically involves a multidisciplinary team that delivers a range of high-intensity psychiatric interventions. It provides an alternative to inpatient hospitalization for individuals experiencing acute psychiatric decompensation (1–3). This model was proposed as a consequence of the deinstitutionalization movement in the 1950s, which represented a structural shift in mental health care aimed at replacing long-term psychiatric hospitals with community-based services to support recovery and promote social integration (4, 5). Psychiatric rehabilitation comprises a person-centered and contextual framework aimed at helping individuals develop stress management skills and improve interactions with their environment (6, 7). Following rehabilitation principles, the community-based, multidisciplinary, and tailored approach of psychiatric home care is a valuable initiative, aiming to improve treatment outcomes, enhance functioning, and support recovery through an individualized therapeutic plan and shared decision-making (8).

Mental health care in Spain is coordinated by the National Health System (NHS). It operates through a decentralized model, working in collaboration with primary care, community mental health centers, day hospitals, rehabilitation services, and a network of inpatient units for acute, subacute, and long-term care (9, 10). Currently, the NHS’s mental health priorities focus on strengthening the primary care network and developing a national mental health strategy based on an integrated community model that incorporates a gender perspective. Its goals include addressing the social determinants of mental health, combating stigma associated with mental illness, promoting community-based care and shared decision-making, and supporting the role of informal caregivers (9). The mental health strategy was developed in response to increasing awareness of national mental health trends and the evolving needs of the population. In 2022, 17% of the Spanish population experienced mental health issues, with a notable gender gap: 22.1% were women compared to 12.1% men. Depression and anxiety were the most common diagnoses in 2021, and Spain ranked as the second-highest consumer of prescribed anxiolytics in the European Union that year (9, 11).

The community-based model has supported the development of various services, including psychiatric home care. These approaches were initially introduced gradually across Spain (12–14). In 2017, the Department of Health of the Government of Catalonia took steps to improve mental health care within community settings (15). A key initiative was the launch of the Primary Support Program (PSP), which places mental health professionals in primary care settings to encourage integrated and collaborative treatment (16). At the same time, the department promoted the expansion of home-based mental health care services, helping to extend the reach of the home care model throughout the region (17–19). In Europe, psychiatric home care has been implemented and evaluated in diverse ways and contexts, with some of the most extensive and well-established developments observed in Northern Europe (20–22), and the United Kingdom (UK) (2, 23–25). In Southern Europe, Spain is the only country to have documented both its experience and preliminary evaluations in this area (17, 18, 26).

A central aspect of psychiatric home care is effective crisis management and person-centered care that is tailored to the individual’s context. Results from previous evaluations suggest that this approach strengthens users’ social networks and fosters a bond of trust among all involved parties, thereby reducing stress and stigma (3, 27). It also helps preserve daily routines, is well accepted by both users and the community (27, 28), and facilitates the identification of risk factors associated with potential readmission to standard inpatient hospitalization (29). Users value respect, recognition of the urgency of their situation, and individualized support (30). Flexibility and reliability are also appreciated, along with the inclusion of family members in the recovery process. Establishing a supportive relationship is often viewed by professionals as requiring strong alignment between care teams, support services, and service users (31).

As in studies conducted in the UK and other European countries, qualitative research in Spain has shown high levels of user satisfaction, attributed to staff professionalism, accessibility and provision of personalized, high quality nursing care (17). However, only user and nurse perceptions were assessed (17, 32), and neither multidisciplinary professional team perceptions nor the impact on service users and their relatives were explored. This study aimed to understand the experience of a psychiatric home care program in Catalonia from the perspective of all those involved.

## 2 Materials and methods

### 2.1 Setting

The Parc Sanitari Sant Joan de Déu (PSSJD) healthcare network has three components: a regional referral hospital offering inpatient/outpatient pediatric and adult care, an intermediate care center for geriatric care, and a Mental Health Network. This Network integrates acute, sub-acute and long-term psychiatric hospitalization and community care, and includes nine Community Mental Health Centers (CMHC) and seven Psychosocial Rehabilitation Centers providing outpatient care for adults with mental health diagnoses. These CMHCs collectively serve a reference population of more than 700,000 individuals in southern Barcelona, Spain (33).

### 2.2 Crisis Resolution and Home Treatment

The Crisis Resolution and Home Treatment (CRHT) team, a psychiatric home care program, was created at PSSJD in 2019 as an alternative to traditional acute inpatient hospitalization. Home-based medical care for people experiencing acute clinical decompensation is provided by a multidisciplinary team including nurses, psychiatrists, psychologists, and social workers with experience in high-intensity psychiatric interventions. The team ensures round-the-clock care (7 days a week, 24 hours a day) for up to 21 days with daily visits. During non-working hours, between 6 p.m. and 8 a.m., users and caregivers have direct telephone access to an emergency psychiatrist. There are two CRHT teams, one serving the Garraf CMHC population and another serving the Cornellà and Esplugues CMHC populations, all in the Barcelona area.

CRHT referrals come from PSSJD emergency or acute care departments, CMHCs, and day hospitals. Eligibility criteria include specific psychiatric diagnosis (e.g. depressive disorders, mania/hypomania, early psychosis, and psychotic disorders without significant secondary behavioral symptoms), linkage to local mental health resources, residing in the catchment area, and presence of an informal caregiver. Exclusion criteria include lack of family support, serious somatic conditions or behavioral disturbances, imminent suicide risk, eating disorders, and substance dependence as a primary diagnosis. Upon discharge from the CRHT, individuals may be referred for outpatient follow-up, day hospital or acute inpatient hospitalization, if needed.

### 2.3 Research team

The research team comprised of six female researchers: MRV (PhD, pharmacist), MGG (PhD, pharmacist), AMB (MSc, pharmacist), MAM (MSc, nurse), LBH (MSc, psychiatrist) and JS (person with lived experience of mental health services). MGG and AMB are full-time researchers; MRV combines research with managing the Quality and Patient Safety Unit at PSSJD. MAM works full-time in a CRHT team, and LBH is deputy director in mental health with extensive clinical experience. JS has led community recovery groups. MGG and MRV, experts in qualitative research, mentored AMB, a predoctoral researcher. MAM and LBH had prior qualitative research experience; JS did not. The research team had no prior relationship with the study participants, except for MAM, who may have interacted with some of the users and caregivers through her work with the CRHT team. MAM was also interviewed as a member of the CRHT team.

### 2.4 Study design and participants

We conducted a qualitative study using a phenomenological approach to explore experiences with the CRHT program, in light of the limited literature available in Catalan and Spanish contexts. Theoretical and convenience sampling were used to recruit participants. Participants consisted of three groups: (1) CRHT service users, with the inclusion criterion that they had been discharged prior to study participation, (2) family members and/or caregivers of CRHT users, and (3) health professionals from CRHT teams. No exclusion criteria were applied. The historical user list was obtained by the person responsible for CRHT administrative tasks, and one researcher contacted participants by phone or email, aiming to achieve the greatest possible diversity in gender, age, education level, and diagnosis.

Following an early interview, a treating psychiatrist suggested that the study interview may have interfered with the user’s recovery. Thus, for subsequent interviews, the user’s health status and suitability for participation was first confirmed with the treating psychiatrist prior to study invitation. From this point onwards, it was considered an inclusion criterion for the study. Four users declined to participate due to lack of interest. Caregivers were contacted with users’ permission, and later invited to participate directly by CRHT staff. All CRHT professionals participated in the study, and team coordinators were responsible for organizing interviews directly with professionals and researchers.

### 2.5 Data collection

Data were collected between April 2021-April 2022 in semi-structured face-to-face individual and group audio-recorded interviews. CRHT users were interviewed individually by a researcher (AMB) at home or at a location of their choice. Caregivers were interviewed in groups rather than individually, as initially planned, given participant access and time restrictions. CRHT team members were interviewed collectively, with individual interviews scheduled for those who could not attend. Group interviews were conducted at the CMHC with one researcher (AMB) facilitating and another (MGG) observing.

CRHT-user interviews followed a thematic guide exploring accessibility, information, shared decision-making, expectations, family involvement, and relationships with professionals. Caregiver interviews also examined the experience of caring for a loved one in a mental health crisis and CRHT support. Interviews with professionals also covered CRHT organization and functioning, including inter-professional collaboration and team dynamics. The interview guide was developed based on CRHT admission stages, and findings from qualitative studies in psychiatric home-based care. It was reviewed by clinicians with experience in psychiatric home hospitalization. No modifications were deemed necessary following initial interviews. A field journal was kept throughout the study, in which the interviewer (and, for group interviews, the observer) documented field notes along with additional insights that aided case contextualization. Following each interview, the information collected was summarized and participant feedback was sought to validate interpretation and allow participants to correct and add to the information as needed. None of the interviews had to be repeated. Recruitment and data collection continued until data saturation was reached (defined as the point at which additional data no longer generated new themes or information relevant to the research question) (34).

### 2.6 Data analysis

A thematic analysis of collected data was performed in accordance with Braun and Clarke’s recommendations (35): become familiar with the data, generate initial codes, search for themes, review themes, define and name themes and produce the final report. Researchers manually transcribed, pseudonymized, and reviewed the audio-recorded interviews. The transcripts were then read and reread, individually coded, and triangulated between AMB, MGG, and MAM. The team inductively identified themes through a reflective process, comparing and agreeing on meanings and creating initial categories. The results were reviewed by two members of the research team: JS, a mental health service user from PSSJD who was not interviewed, and LBH, an experienced CRHT psychiatrist. Atlas.ti 22 software was used to support data management.

### 2.7 Ethical considerations

As this study was part of a quality improvement project by the PSSJD Quality and Patient Safety Unit, it did not require evaluation by the research ethics committee. Nevertheless, following institution internal protocols, important ethical aspects were considered at all stages using a checklist: project scope; involvement of the individual, family/caregivers and healthcare personnel; informed consent; access to information of individuals and family/caregivers; and potential harms. The authors assert that all procedures contributing to this work comply with the ethical standards of the relevant national and institutional committees on human experimentation and with the Helsinki Declaration of 1975, as revised in 2013.

Given the sensitive nature of the personal data collected in this project, we ensured compliance with the European Union General Data Protection Regulation (GDPR) through the following measures: (1) Obtaining explicit informed consent. Participants received verbal and written details on the study, its aims, and what their involvement entailed, including details on the type of data collected (audio recording of the interview), how it would be stored and protected, who would have access to it, how long it would be retained, and their rights to access, correct, or delete their personal data. All participants signed an informed consent form, confirming their voluntary participation. (2) Ensuring confidentiality. Interviews were pseudonymized at the time of transcription, and each participant was assigned a unique code. (3) Securing data. All data were stored on encrypted devices, with access restricted to authorized personnel only, and potential risks of participant identification (e.g., in the case of a potential data breach) were minimized through early pseudonymization and removal of identifying information from the processed data.

### 2.8 Techniques to enhance trustworthiness

This study used Guba and Lincoln’s trustworthiness criteria, which include credibility, transferability, dependability, and confirmability (36). Credibility was supported by the researcher’s in-depth understanding of the organizational and structural aspects of CRHT programs. This knowledge was passed onto the research team members in a comprehensive briefing. Furthermore, triangulation of findings was conducted by researchers from different disciplines and experiences, and feedback from interviewees provided on interview completion made it possible to corroborate the interpretation of the information.

Transferability was ensured by the theoretical selection of the sample and the detailed description of the data set and how it was obtained. Dependability was achieved through a review of the project by a team of external researchers, experts in qualitative research, in the Committee for Qualitative Health Research, coordinated by the “Consorci de Salut i Social de Catalunya” (CSC) and the “Institut Universitari d’Investigació en Atenció Primària” (IDIAPJGol). The study objectives and methodology were presented to this committee, which provided an opportunity for reflection and support for methodological validity. Confirmability was achieved by reflecting on individual researchers’ subjective viewpoints in relation to the study, and by presenting an update on preliminary results and methodological modifications made to the project to the “Committee for Qualitative Health Research”, for discussion. The Consolidated Criteria for Reporting Qualitative Research checklist was used (37) (**Supplementary File**).

## 3 Results

Thirty-five people participated in the study, including 11 users, 10 caregivers and 14 CRHT team professionals. The gender, age, and educational level of the CRHT users varied widely, and most caregivers were parents of users and in employment. There was a high proportion of very experienced nurses and professionals (between 11-20 years of experience) and most identified as female (**Tables 1**, **2**). We conducted 14 individual interviews (11 with users and three with professionals; average duration=38 minutes) and four group interviews (two with caregivers and two with professionals; average duration=84 minutes).

**Table 1:** Sociodemographic characteristics of CRHT service users and caregivers.

Sociodemographic characteristics		Service users *N=11*	Caregivers *N=10*
Gender		*N* (%)	*N* (%)
	Female	6 (54.5)	6 (60.0)
	Male	5 (45.5)	4 (40.0)
Age		*N* (%)	*N* (%)
	18 – 30	3 (27.3)	0 (0.0)
	31 – 40	2 (18.2)	0 (0.0)
	41 – 50	3 (27.3)	3 (30.0)
	51 – 60	2 (18.2)	2 (20.0)
	61 +	1 (9.0)	5 (50.0)
Education		*N* (%)	*N* (%)
	None	2 (18.2)	0 (0.0)
	Primary school	1 (9.0)	2 (20.0)
	Secondary school	5 (45.5)	7 (70.0)
	Higher education	3 (27.3)	1 (10.0)
Employment		*N* (%)	*N* (%)
	Student	1 (9.0)	0 (0.0)
	Working	2 (18.2)	8 (80.0)
	Work leave	1 (9.0)	0 (0.0)
	Pensioner	7 (63.8)	1 (10.0)
	Retired	0 (0.0)	1 (10.0)
Nationality		*N* (%)	*N* (%)
	Spanish	9 (81.8)	9 (90.0)
	Other	2 (18.2)	1 (10.0)
Cohabitation		*N* (%)	*N* (%)
	Alone	4 (36.4)	0 (0.0)
	Mother and/or father	4 (36.4)	0 (0.0)
	Partner	1 (9.0)	3 (30.0)
	Partner and children	2 (18.2)	4 (40.0)
	Children	0 (0.0)	2 (20.0)
	Other relatives	0 (0.0)	1 (10.0)
Relationship to service user		*N* (%)	*N* (%)
	Mother/father	N/A	6 (60.0)
	Partner	N/A	1 (10.0)
	Ex-partner	N/A	1 (10.0)
	Sister	N/A	1 (10.0)
	Son	N/A	1 (10.0)

CRHT, Crisis Resolution and Home Treatment; N/A: Not applicable..

**Table 2:** Sociodemographic characteristics of CRHT team professionals.

Sociodemographic characteristics		Team professionals *N=14*
Gender		*N* (%)
	Female	12 (85.7)
	Male	2 (14.3)
Age		*N* (%)
	18 – 30	4 (28.6)
	31 – 40	6 (42.8)
	41 – 50	4 (28.6)
Credentials		*N* (%)
	Nurse	7 (50.0)
	Psychiatrist	3 (21.4)
	Psychologist	2 (14.3)
	Social worker	1 (7.1)
	Administrative tasks	1 (7.1)
Years of experience		*N* (%)
	0 – 5	4 (28.6)
	6 – 10	3 (21.4)
	11 – 15	4 (28.6)
	16 – 20	2 (14.3)
	20 +	1 (7.1)

CRHT, Crisis Resolution and Home Treatment.

**Figure 1:**
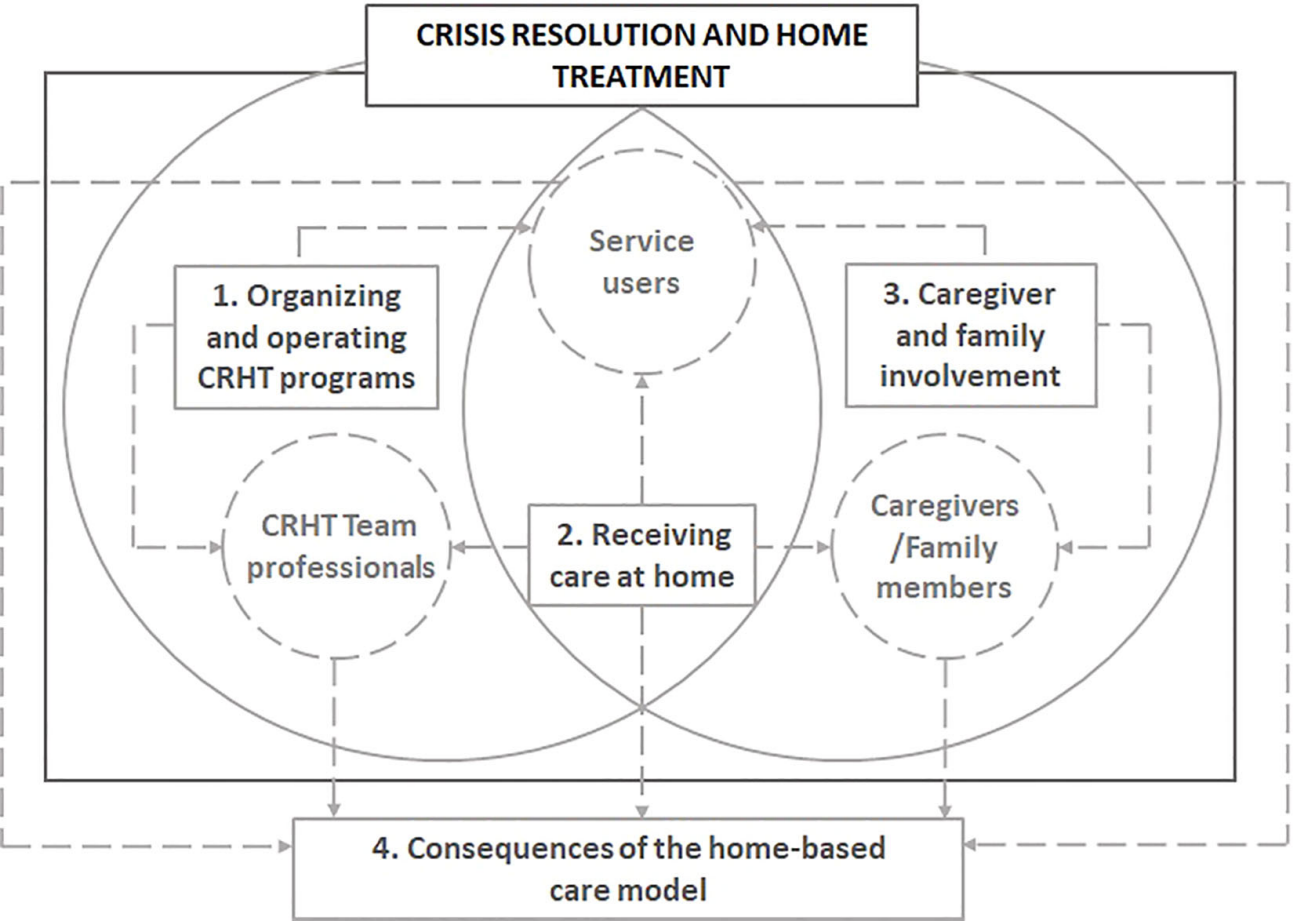
Relationship between major themes and study participants.

Four main themes summarized CRHT-program experiences: (1) Organizing and operating CRHT programs; (2) Receiving care at home; (3) Caregiver and family involvement and (4) Consequences of the home-based care model. The themes are described below, with narrative quotes to support the data. Service user data are indicated by SU1-11, family caregiver data by FC1–10 and healthcare professional data by HP1-14. **Figure 1** summarizes the relationship between major themes and study participants. **Table 3** presents the list of themes and sub-themes, identifying the profile of participants that expressed views on them.

**Table 3:** Main themes and sub-themes emerged from the interviews, according to participants.

Themes (sub-themes)	Participant
Organizing and operating CRHT programs^a^	
Previous expectations	HP
Multidisciplinary work	SU, FC, HP
Access to CRHT	SU, FC, HP
Initial information	SU, FC, HP
Assessment	HP
Intensity and timeliness of visits	SU, FC, HP
Continued care after discharge	SU, FC
Receiving care at home^b^	
Safe environment	SU, FC
Socialization and mutual support	SU, HP
Personalized treatment	FC, HP
Bed flexibility	HP
Therapeutic bond	SU, FC, HP
Suitability	SU, FC, HP
Caregiver and family involvement^c^	
Caregiver selection	SU, HP
Caregiver’s awareness	FC, HP
Family bonding	FC
Gratitude	SU
Caregiver involvement	SU, FC, HP
Support for family members	FC, HP
Consequences of the home-based care model^d^	
Person-centered care	SU, FC, HP
Long-term intervention	HP
Shared decision-making	SU, HP
Empowerment	SU, FC, HP
Accompanying	SU, HP
Reducing geographic barriers	SU, FC, HP
Insight and health improvement	SU, FC, HP
Adherence	FC, HP
Self-stigma	HP
Burden of care and self-care	SU, FC, HP
Role of CRHT	SU, FC, HP
Professional satisfaction	HP

^a^Experience in organizing and running the CRHT programs to date; ^b^Effect of the care model at the user’s home; ^c^How the presence of the caregiver and/or family may affect admission; ^d^Impact of home-based care on all those involved.

CRHT, Crisis Resolution and Home Treatment; FC, Family caregiver; HP, Healthcare professional; SU, Service user.

### 3.1 Organizing and operating CRHT programs

Before working in a CRHT team, crisis management in a nontraditional hospital setting was a source of uncertainty for professionals. Treating a person in crisis without 24-hour observation was hard for them to imagine*; “At first, right? Can we deal with this type of patient at home? And the fears they produce in the professionals, not having the patient monitored for 24 hours, when they are in a crisis”* (HP3).

They also noted that CRHT allows for truly multidisciplinary work, which is not always achieved in other work settings. It strengthens team bonds, transforms the workplace into a shared and trusted space, and allows professionals to learn from other perspectives and disciplines, broadening their skills through cross-functional collaboration; *“I’ve never made visits with a social worker and a psychologist and I’m learning a lot from other disciplines. And this gives me far more resources, doesn’t it? I mean, for the patients”* (HP2). However, professionals felt unaccompanied at weekends as visits are one-to-one.

#### 3.1.1 Access to CRHT

For some users, recalling referral and admission was difficult, although most felt the process was quick; *“They processed it and I had a physician at home in two days. I mean, it was super-fast”* (SU7). They stated that CRHT information and communication around it was very reassuring to them and their caregivers, especially for users with dependents (e.g., children) or those unfamiliar with the program; *“When they noticed I was so nervous, they explained in a very natural way that it wasn’t anything bad, that I was in a program, that I would have my baby with me (…) They make you feel calmer”* (SU2). Professionals felt that using visual materials and spending more time on the initial interview would improve information provision; *“Probably a leaflet or something that you, the family, could, from here, say “Listen, what happens if I can’t be here all day?” or “How do we manage, if I have a question?”* (HP14).

Upon referral, the CRHT team conducted an initial eligibility-confirmation assessment. Program inclusion criteria were sometimes relaxed, for example, when referral services were saturated, or when formal caregivers were unavailable but professionals considered users to be fully capable of self-care; *“Our unwritten internal algorithm would be: we accept that there is no caregiver if it is very clear that there aren’t any other social issues”* (HP9). Even though it was an exclusion criterion, professionals and users were concerned about the risk of self-harm. After a suicide during a CRHT admission, the team was worried about future incidents and had a heightened sense of insecurity; *“For instance: suicide risk. It’s very difficult to assess. Very difficult to foresee. We had a completed suicide [of a user] in the team, which also made us worry about the issue”* (HP9).

#### 3.1.2 Home hospitalization and continuity of care

Being punctual and warning of late arrival was appreciated. Visiting hours were flexible and could be tailored to individual needs, but in some cases there was no schedule, disrupting individuals’ appointments; *“(…) you can come to the Rehabilitation Service and then do the CRHT, lots of people do that”. So, of course, I said: “Yes, but they don’t tell me when they’re coming, so I have to wait at home”* (SU6). According to professionals, the frequency and intensity of visits and calls are adapted to each person’s needs, with greater intensity initially; *“…we try to adapt to the patient’s needs. The assessments, the initial visits, we always dedicate more time”* (HP1). In general, users and caregivers considered visit frequency and duration adequate, except for a few who preferred longer visits and one who wanted less frequent or remote visits; *“Well, with more time. With videoconference, videocalls, I mean, do the follow-up in other ways that are more comfortable. For them and for me”* (SU5).

Users and caregivers would prefer longer CRHT stays and felt unprepared for discharge; *“But perhaps, the duration, a little longer, well yes, because … Because I felt I wasn’t back to myself, you know?”* (SU9). This could be due to the high intensity of care provided by CRHT compared to post-discharge community services, which may leave users feeling helpless; *“…suddenly it stops. It stops on Monday and on Tuesday you don’t have anyone (…) so it creates a bit of a vacuum effect”* (SU8).

### 3.2 Receiving care at home

Users saw their home as a safe, comfortable, protected environment. It gives them peace of mind and allows them to continue their daily routines and freely determine their schedules and dynamics; *“…the well-being I felt when I did CRHT or now, in my free time, I can, I don’t know, shower in half an hour, eat in an hour and a half, whatever … lie down on the couch and watch a movie”* (SU4). Having the entire CRHT team at home could be overwhelming for some people, especially at the beginning. However, the home provides structural comforts not available in hospitals, and users reported feeling safer at home during crises, especially because they avoid exposure to others with symptoms that may interfere with their recovery; *“You’re not somewhere with other patients who have other illnesses that you can catch. In the sense that you see yourself so you start to think and then start to say: “Hang on! This could happen to me!”* (SU6). While most users preferred to manage a mental health crisis at home, some with a history of hospitalization or inadequate support networks valued the traditional inpatient setting for socialization and peer support, even seeing it as a home away from home; *“Admitted or at home, more or less, for me, it’s nothing really. Even more pleasant there because I* sp*oke with more people, with the sick and so on”* (SU3).

In the user’s home, professionals can evaluate the case comprehensively, observing the individual’s family and daily environment, with longer visits than in traditional hospitalization. It objectifies the user’s reality in a more global way, facilitating treatment plan design; *“…the approach is more individualized at home, I have more time to give them. I can see what their environment is like, I can see what the family is like, how he/she functions at home. Things I can’t see in the hospital”* (HP12). Additionally, since space and beds are not physically limited, teams can be organized to increase the number of beds as needed; *“If we are at our maximum and if we have to add an extra bed one day, two days … If you find yourself in that situation, it can be done”* (HP3).

All participants agreed that working in the user’s context fosters a close, trusting, non-hierarchical relationship between professionals, users and family members, creating a strong bond. In some cases, this bond is maintained and even continues beyond the CRHT admission; *“It’s very important, with mental illnesses, that an emotional bond grows, a wonderful bond, a warm bond, between the therapists, any of them, and the patient (…) And with CRHT a truly delightful bond is established”* (SU4). However, despite the benefits of being at home during a crisis, all participants recognized that home hospitalization is not appropriate for all users and depends on each person’s clinical condition and situation; *“It depends on the patient (…) Some are more well-disposed to it than others, right? And the context too”* (HP3).

### 3.3 Caregiver and family involvement

The caregiver role can be played by any trusted person capable of taking responsibility for home care, and the choice of caregiver is agreed between the team and the user according to their preferences; *“In fact, the user decides. Who do they want to be their go-to person, based on trust, who they feel most comfortable with?”* (HP5). In this study, all caregivers were family members.

To help caregivers feel confident and at ease, the team is responsible for providing them with necessary information about the user’s health and treatment. In addition, caregivers felt that active participation in their family member’s treatment increased their awareness of their role and responsibility in the recovery process compared to their experiences before CRHT; *“…this team is not alone, this team works because the family is around. If not, it wouldn’t work.”* (FC8). By being a part of the experience, family members can better understand the process and empathize with their loved one. Furthermore, CRHT fosters a close bond between users and their families, strengthening ties and mutual understanding; *“When you have him at home, on the other hand, it’s a relief because you see how they’re improving every day, how they’re improving. Of course, you get closer to them and they get closer to you”* (FC2). For users, feeling accompanied and supported by their family and loved ones while recovering created a strong sense of gratitude; *“Very lucky. Very grateful. Because they were there for me and I felt that love and support”* (SU9).

Caregiver involvement was personalized to meet user and caregiver preferences and interests. In some cases, they liaised with the team, providing information that the user preferred not to comment on directly; *“Because the girl* sp*oke to her about things I didn’t tell them. So, she went, I talked to her, my wife, about things, well, that I, worried about, what made me feel bad, or whatever”* (SU11). In other cases, caregivers were excluded from visits if they were felt to be barriers to recovery or if the user preferred to keep their symptoms or health status confidential; *“When they asked me about my symptoms and so on, my mother wasn’t there. Because, I think … They are a little grim. So I didn’t want her to worry”* (SU10).

Professionals attempted to provide caregivers with tools that would be helpful in managing the user’s crisis episodes, especially in cases where the family was involved for the first time. Still, it could be shocking for users’ families to experience the acute phase of the illness firsthand, and in some cases they may need additional resources to bear and manage the crisis. This was especially true of relatives with chronic mental health problems and families with young children; *“And me? I am here too. I also suffer it. And not for myself, for my daughter who is seventeen years old and has been suffering with it since she was born”* (FC6).

### 3.4 Consequences of the home-based care model

The CRHT team provides comprehensive care, addressing the crisis in a holistic manner, considering employment, housing, socialization, and family relationships to promote person-centered care. The team always adapts care to each individual’s needs and preferences, which is perceived as more sensitive and humane than traditional hospitalization; *“Then the psychologist would always want to go for a walk (…) she always wanted us to get some sun. Of course, I never left home, with depression you’re on the couch all day and she made me get dressed and … let’s go!. let’s get a coffee or go for a walk!”* (SU4). According to CRHT professionals, a comprehensive crisis approach allows for the development of long-term treatment plans coordinated with community resources more easily than traditional inpatient treatment; *“And the interventions are much more long-term, at the time you’re doing them, in your normal surroundings, aren’t they? An intervention that you can do in the hospital, it has no continuity”* (HP3).

Whenever possible, the team promotes shared decision-making based on individual preferences, including agreement on medications, visitation frequency and family involvement; *“Both, I think it was by mutual agreement. But it started with them, because the psychiatrist too, I suppose he realized I was getting better and I didn’t have to take the medication for so long”* (SU2). They also prepare users and their families to manage their care after discharge by providing information about health issues and treatments, which promotes empowerment; *“…fostering the patient’s autonomy and that they understand their therapeutic process, know what it entails, know about the illness, know all about their disorder. Both them, and the family”* (HP10).

Participants appreciated having professionals available during CRHT (a team member or psychiatrist was always reachable), either at home or on the phone after-hours. This uninterrupted access was seen by professionals and users as continuous care, reassuring and comforting users, even providing a sense of well-being; *“…I felt that I wasn’t alone. Because sometimes I feel, due to my illness, very alone and I felt more accompanied. Whether you want it or not, it helps you”* (SU10).

Providing care at home is more complicated for professionals as they have to travel. However, barriers to access for both the user and the family are reduced by bringing care closer to the community; *“…there’s no queueing, you don’t have to make appointments, I mean, they come to you and there they are, and there they are”* (SU4). Users and caregivers also perceive good health and functional recovery and improved awareness of their condition and its severity; *“He now accepts that he is sick. There was even a moment when he said “I want them to admit me, I’m not well”. And before, no”* (FC5). CRHT was also associated with improved treatment adherence, although practitioners noted that family involvement could occasionally be a barrier to adherence if not aligned with clinical goals; *“Of course, sometimes we have encountered uncooperative families, right? I would say”* (HP14). Professionals also commented that self-stigma is reduced because users cannot compare themselves to others, although this can result in skewed perceptions of their illness severity. In addition, they have the opportunity to work together on strategies to reduce it; *“…you can work a bit on stigma. You eliminate false beliefs or learned beliefs that, with psychiatry, it’s easier to break down barriers like this”* (HP13).

Professionals noted that for some, particularly women, home care responsibilities and caring for other family members can interfere with recovery and even privacy during visits; *“A mother will always have the kids and if they are small … then it means respecting* sp*aces a little. Sure, if she were at the hospital, it would be just her during the visit. She would have her privacy”* (HP13). It can also place an additional burden on caregivers, who often try to juggle the demands of work, home, and routines on top of caring responsibilities; *“…because we were in lockdown. If not, I could not have been the caregiver (…) the only income is from my job, so, I have to work”* (FC1).

The important role of CRHT as an alternative to inpatient mental health crisis care, avoiding or reducing hospital admissions, was seen by all participants; *“Positive. Yes. You see it as a ray of light, don’t you? Because, of course, between going to the emergency room and being admitted, it’s an intermediate point”* (SU3). Professionals also reported satisfaction with therapeutic/professional relationships, more time for care, and greater user improvement and recovery; *“You see the whole improvement process, don’t you? And I think this is very satisfying at the professional level”* (HP8).

## 4 Discussion

Our qualitative findings suggest that CRHT promotes multidisciplinary work, is helpful for adapting processes and treatment to each individual, strengthens interprofessional relationships, and creates stronger bonds between professionals, users, and families. Participants rated home care highly and their perception of safety was good, although some still preferred conventional psychiatric admission. Post-discharge, users and caregivers felt unprepared to cope due to the significant reduction in care intensity. Caregivers were aware of their responsibilities, and users appreciated their family member’s care, and although experiencing a loved one’s recovery strengthens family bonds, some caregivers may need additional support to manage crises. Along with personalized care, CRHT allows for the development of a comprehensive, lifelong treatment plan, although the burden of care and self-care must be considered, especially when caring for other family members.

To date, this study is the first to explore service user, caregiver and professional experiences of CRHT teams in Spain. Descriptive studies and nurse/service user experiences have been reported independently, but not as a shared experience of all stakeholders (12, 13, 17, 18, 26, 32). Consistent with other studies, referral to the CRHT was perceived as a positive experience, especially in terms of admission criteria flexibility, detailed information on processes and reassurance of users and families (17, 31). Professionals should consider user/family expectations at referral and the team’s capacity to meet them (38). Regarding exclusion criteria, suicide risk remains difficult to detect in initial assessments. Witnessing medical safety events, especially self-harm or suicide during psychiatric home care, has a major impact on staff as well as on subsequent assessments of the service user. Staff are at a high risk of experiencing the second victim phenomenon, leading to negative emotions, lack of trust, guilt, and frustration (39, 40). These effects may directly impact quality of life and working conditions.

Despite professionals’ global perspective and coordination with other services, transition to a mental health resource after CRHT is frequently a difficult experience for users and families due to care fragmentation among the various services. Reducing end-of-care visits and promoting inter-service coordination is critical to ensuring continuity of care and preventing users from feeling abandoned (31, 41). Although these strategies are being implemented, users still perceive discontinuation of care as a major problem.

According to Winnes (42), for some, psychiatric ward hospitalization may be preferable as it separates the person from the crisis context (i.e., home) and promotes social interaction with peers. However, for most users, home is perceived as a comfortable, safe place to recover, and research suggests that the provider-user relationship is less hierarchical in the home setting (17, 31, 43). Individuals and families are more horizontally and equitably connected to professionals, which also increases sensitivity and awareness of the therapeutic relationship. Furthermore, the home environment favors understanding of users’ rights and needs, shared decision-making regarding treatment, and users’ and family members’ satisfaction with daily visits (31, 44).

Caregiver burden must be carefully assessed. Home care has not been shown to reduce overall caregiver burden, and although initiatives exist regarding daily household tasks and emotional support, they are not generally perceived as adequate by family members (31, 42, 45). The same holds for female users with dependents, especially those with young children, where the home visit may interfere with daily tasks, or prove intrusive and overwhelming (25). In this study, and in previous experiences evaluated in similar settings (31, 46), CRHT teams were found to play a fundamental role in liaison between hospital and community mental health services, and comprehensive care provision, although this aspect of CRHT should be further explored.

This study has some limitations. While the qualitative approach and reflexive thematic analysis methodology allowed for an in-depth approach and data-familiarity (35), it is likely that the sample does not reflect the experiences of service users who are less engaged with mental health services. It is common, especially in service evaluation studies, for users (and in this case, also caregivers) who are more satisfied with the service provided to be more motivated to participate. In this study, we did interview individuals who were dissatisfied with the service provided by the CRHT teams; however, they represented a minority compared to those who expressed satisfaction. Another limitation of the study concerns the process of accessing the sample. For caregivers, direct recruitment by CRHT professionals may have influenced participation, as they may have felt a sense of obligation out of gratitude for the service provided. However, the range of responses collected suggests that caregivers nevertheless felt able to express both positive and critical views. The group interviews conducted with professionals included the entire CRHT team. This means that team coordinators were also interviewed as part of the group. This situation may have influenced participants’ responses, as they might have moderated their answers due to the hierarchical relationship with other members of the interview group. Despite potential power dynamics influencing group discussions with professionals, rich discussions were generated and subsequent conversations with individual staff members to corroborate findings did not raise any concerns about this. Finally, the setting in which the evaluation was conducted corresponds to a specific and unique context, characteristic of the geographical area covered by the CRHT teams. Even though the evaluation focused on this very particular setting and therefore the results are not generalizable, our aim was in fact not generalizability, but rather, to explore this specific context and situation.

CRHT appears to be a valuable initiative, showing consistent stakeholder satisfaction and contributing to more efficient use of healthcare resources (19). Its integration within the community-based mental health care system not only facilitates access to services but also strengthens trust in healthcare professionals. These observed benefits suggest that the model could be a promising option for implementation in other regions of the country, where it could help improve both service delivery and user experience. Exploring and understanding the lived experience of everyone involved with CRHT teams has proven to be an opportunity for learning and professional growth for its members and a comfortable, safe and accompanied way for users and their families to overcome a mental health crisis. It also presents an opportunity to design and implement future strategies to improve patient care, always considering a collaborative approach that takes into account the opinions and experiences of all stakeholders. In this case, opportunities could be directly aimed at improving the continuity of care between CRHTs and community resources, as well as the creation of a support plan that considers the real and particular needs of users and their families, whether practical, relational, or emotional. It remains crucial to further study the referral processes to and from the CRHT programs in depth and to propose, from a participatory approach, the establishment of common processes that improve communication, information and mutual understanding between the different mental health resources and all the people involved.

## 5 Conclusions

This CRHT program was positively valued by participants as a flexible intervention that facilitates person-centered care and strengthens therapeutic bonds. Home-based care enables professionals to tailor treatment to individual needs, foster non-hierarchical relationships with users and their families, and enhance clinical insight. In addition, family caregiving in the perceived safety of the home strengthens the bond with the service user, although additional support needs should always be assessed. Care and self-care burden on the user and family must also be taken into account, especially as home hospitalization may not be appropriate for all.

As the first known study in Spain to explore CRHT from a multi-stakeholder perspective, our findings can be used to develop quality improvement tools for CRHT in other contexts and populations (particularly regarding continuity of care, and service coordination) to promote a community-based model of mental health care across the lifespan.

## Data Availability

The raw data supporting the conclusions of this article will be made available by the authors, without undue reservation.
