# Correction: Exploring lived experiences in home-based psychiatric care: a qualitative study of service users, families, and professionals in Spain

**DOI:** 10.3389/fpsyt.2026.1783795

**Published:** 2026-01-16

**Authors:** Ana María Besoaín-Cornejo, Montserrat Gil-Girbau, Mariam Alouali-Moussakhkhar, Luisa Baladón Higueras, Josefina Sáez, Maria Rubio-Valera

**Affiliations:** 1 Health Technology Assessment in Primary Care and Mental Health (PRISMA) Research Group, Teaching, Research & Innovation Unit, Institut de Recerca Sant Joan de Déu, Esplugues de Llobregat, Spain; 2 Network for Research on Chronicity, Primary Care and Health Promotion (RICAPPS), Instituto de Salud Carlos III, Madrid, Spain; 3 Facultat de Farmàcia i Ciències de l’Alimentació, Universitat de Barcelona (UB), Barcelona,, Spain; 4 Parc Sanitari Sant Joan de Déu, Sant Boi de Llobregat, Spain; 5 Patient Research Partner, Sant Pere de Ribes, Barcelona, Spain; 6 Center for Biomedical Research Network in Epidemiology and Public Health (CIBERESP), Instituto de Salud Carlos III, Madrid, Spain; 7 Medicine and Life Sciences, Universitat Pompeu Fabra, Barcelona, Spain

**Keywords:** crisis management, health services research, mental health, patient-centered care, qualitative research

An incorrect **Funding** statement was provided. The correct funder is “Instituto de Salud Carlos III (ISCIII) under the program of Cooperative Research Projects Oriented to Health Outcomes (RICORS) 2021, with funds from the European Recovery, Transformation and Resilience Plan (PRTR) and the National Agency for Research and Development (ANID).”

The correct funding statement reads:

“The authors declare financial support was received for the research and/or publication of this article. This work received financial support from the Instituto de Salud Carlos III (ISCIII) under the program of Cooperative Research Projects Oriented to Health Outcomes (RICORS) 2021, with funds from the European Recovery, Transformation and Resilience Plan (PRTR). Program funded by the European Union – NextGenerationEU, grant number RD21/0016/0018. This work was also supported by the National Agency for Research and Development (ANID)/Scholarship Program/DOCTORADO BECAS CHILE (grant number 2019–72200124).”

The original version of this article has been updated.

