# Supplementary material for: Exploring lived experiences in home-based psychiatric care: a qualitative study of service users, families, and professionals in Spain
Source: Front Psychiatry. 2025 Sep 23;16:1670470. doi: 10.3389/fpsyt.2025.1670470 (PMC12500623; doi:10.3389/fpsyt.2025.1670470)
Supplement: Supplementary file 1 [file Table1.docx]

Supplementary Material

**Consolidated criteria for reporting qualitative studies (COREQ): 32-item checklist**

| **Nº** | **Item** | **Guide questions/description** | **Reported in section:** |
| --- | --- | --- | --- |
| **Domain 1: Research team and reflexivity** | | | |
| *Personal Characteristics* | | | |
| 1. | Interviewer/facilitator | Which author/s conducted the interview or focus group? | 2.5. Data collection |
| 2. | Credentials | What were the researcher’s credentials? *E.g. PhD, MD* | 2.3. Research team |
| 3. | Occupation | What was their occupation at the time of the study? | 2.3. Research team |
| 4. | Gender | Was the researcher male or female? | 2.3. Research team |
| 5. | Experience and training | What experience or training did the researcher have? | 2.3. Research team |
| *Relationship with participants* | | | |
| 6. | Relationship established | Was a relationship established prior to study commencement? | 2.3. Research team |
| 7. | Participant knowledge of the interviewer | What did the participants know about the researcher? *e.g. personal goals, reasons for doing the research* | 2.3. Research team |
| 8. | Interviewer characteristics | What characteristics were reported about the interviewer/facilitator? *e.g. Bias, assumptions, reasons and interests in the research topic* | 2.3. Research team |
| **Domain 2: Study design** | | | |
| *Theoretical framework* | | | |
| 9. | Methodological orientation and Theory | What methodological orientation was stated to underpin the study? *e.g. grounded theory, discourse analysis, ethnography, phenomenology, content analysis* | 2.4. Study design and participants |
| *Participant selection* | | | |
| 10. | Sampling | How were participants selected? *e.g. purposive, convenience, consecutive, snowball* | 2.4. Study design and participants |
| 11. | Method of approach | How were participants approached? *e.g. face-to-face, telephone, mail, email* | 2.4. Study design and participants |
| 12. | Sample size | How many participants were in the study? | 3. Results |
| 13. | Non-participation | How many people refused to participate or dropped out? Reasons? | 2.4. Study design and participants |
| *Setting* | | | |
| 14. | Setting of data collection | Where was the data collected? *e.g. home, clinic, workplace* | 2.5. Data collection |
| 15. | Presence of non-participants | Was anyone else present besides the participants and researchers? | 2.5. Data collection |
| 16. | Description of sample | What are the important characteristics of the sample? *e.g. demographic data, date* | 3. Results |
| *Data collection* | | | |
| 17. | Interview guide | Were questions, prompts, guides provided by the authors? Was it pilot tested? | 2.5. Data collection |
| 18. | Repeat interviews | Were repeat interviews carried out? If yes, how many? | 2.5. Data collection |
| 19. | Audio/visual recording | Did the research use audio or visual recording to collect the data? | 2.5. Data collection |
| 20. | Field notes | Were field notes made during and/or after the interview or focus group? | 2.5. Data collection |
| 21. | Duration | What was the duration of the interviews or focus group? | 3. Results |
| 22. | Data saturation | Was data saturation discussed? | 2.5. Data collection |
| 23. | Transcripts returned | Were transcripts returned to participants for comment and/or correction? | - |
| **Domain 3: Analysis and findings** | | | |
| *Data analysis* | | | |
| 24. | Number of data coders | How many data coders coded the data? | 2.6. Data analysis |
| 25. | Description of the coding tree | Did authors provide a description of the coding tree? | - |
| 26. | Derivation of themes | Were themes identified in advance or derived from the data? | 2.6. Data analysis |
| 27. | Software | What software, if applicable, was used to manage the data? | 2.6. Data analysis |
| 28. | Participant checking | Did participants provide feedback on the findings? | 2.5. Data collection |
| *Reporting* | | | |
| 29. | Quotations presented | Were participant quotations presented to illustrate the themes / findings? Was each quotation identified? *e.g. participant number* | 3. Results |
| 30. | Data and findings consistent | Was there consistency between the data presented and the findings? | 3. Results |
| 31. | Clarity of major themes | Were major themes clearly presented in the findings? | 3. Results |
| 32. | Clarity of minor themes | Is there a description of diverse cases or discussion of minor themes? | 3. Results |

Developed from: Tong A, Sainsbury P, Craig J. Consolidated criteria for reporting qualitative research (COREQ): a 32-item checklist for interviews and focus groups. International Journal for Quality in Health Care. 2007. Volume 19, Number 6: pp. 349 – 357.
